# Migration of aluminum from food contact materials to food—a health risk for consumers? Part I of III: exposure to aluminum, release of aluminum, tolerable weekly intake (TWI), toxicological effects of aluminum, study design, and methods

**DOI:** 10.1186/s12302-017-0116-y

**Published:** 2017-04-12

**Authors:** Thorsten Stahl, Sandy Falk, Alice Rohrbeck, Sebastian Georgii, Christin Herzog, Alexander Wiegand, Svenja Hotz, Bruce Boschek, Holger Zorn, Hubertus Brunn

**Affiliations:** 1 Hessian State Laboratory, Am Versuchsfeld 11, 34128 Kassel, Germany; 2 Hessian State Laboratory, Glarusstr. 6, 65203 Wiesbaden, Germany; 3 Institute of Food Chemistry and Food Biotechnology, Justus Liebig University Giessen, Heinrich-Buff-Ring 17, 35392 Giessen, Germany; 4 Institute of Medical Virology, Justus Liebig University, Schubertstraße 81, 35392 Giessen, Germany; 5 Hessian State Laboratory, Schubertstr. 60, 35392 Giessen, Germany

**Keywords:** Aluminum, Exposure, Migration limit, Tolerable weekly intake

## Abstract

**Background:**

In spite of the prevalence of aluminum in nature, no organism has been found to date which requires this element for its biological functions. The possible health risks to human beings resulting from uptake of aluminum include detrimental effects to the hemopoietic system, the nervous system and bones. Aluminum is used in many fields and occurs in numerous foodstuffs. Food contact materials containing aluminum represent an anthropogenic source of dietary aluminum.

**Results:**

As a result of their frequent use in private households a study was undertaken to detect migration of this metal to foodstuffs from drink containers, coffee pots, grill pans, and camping cookware made of aluminum.

**Conclusions:**

An estimate of the health risk to consumers is calculated, based on the tolerable weekly intake (TWI) specified by the European Food Safety Authority of 1 mg/kg body weight for all groups of people. In some instances the TWI is significantly exceeded, dependent upon the food contact material and the food itself.

## Background

Aluminum is the third most prevalent element in the earth’s crust with a proportion of 7.5%. Certain metals such as iron and magnesium are essential for the human organism and maintenance of bodily functions and must be taken up through nourishment. Aluminum, on the other hand is, in spite of its prevalence, not known to have any function in human or animal organisms [1–4]. According to an older study, aluminum is also not essential for any higher plants [5].^1^ The main source of aluminum in the human body is food. Dermal uptake, on the other hand, plays a secondary role, in spite of the fact that deodorants containing aluminum have been repeatedly suggested to be related to an increase in risk of breast cancer [8–10]. Uptake through inhalation is negligible for the general public, although workers who breathe dust containing aluminum over an extended period have an increased tendency to contract pulmonary aluminosis (restrictive lung disease) [11]. Elemental aluminum finds a myriad of uses due to its excellent material properties such as castability in any shape, plasticity, heat conduction, low density, low melting point, oxidative passivation, and suppleness with concurrent toughness. Consequently, it is highly prized as a construction material, in electrotechnical applications, as packing material or as fuel for solid-fuel rockets, to name but a few uses. Aluminum compounds are used as food additives, in cosmetics and as or in pharmaceuticals. In addition, aluminum occurs naturally in foodstuffs [12–16]. Human exposure is divided into two categories, “external contact” and “dietary contact”; examples of these two categories are presented in Table 1.

**Table 1:** Aluminum—external and dietary contact

Examples of external contact	Examples of dietary contact
Construction materials, including alloys (e.g., vehicle construction, aerospace, suitcases, facades, tent construction)	Packaging and containers (beverage and food cans, coffee pots, outdoor cutlery and dishes, coffee capsules, household aluminum foil)
Electrotechnology, including alloys (e.g., electrical conductors)	Nanoparticles in sunscreens
Fuel for solid-fuel rockets (up to 30% Al) and pyrotechnics	Foodstuffs
Pigments for paints (e.g., “silver” bronze paints)	Toothpaste (e.g., AlF_3_: caries prophylaxis)
Metal polish (Al_2_O_3_: paste, suspension in MeOH or H_2_O)	Pharmaceuticals (e.g., heartburn medicines—pH-regulation; vaccine adjuvants)
	Vaccine adjuvant—(increases the immune reaction)
Organic syntheses (e.g., LiAlH_4_: reducing agent)	Cosmetics (e.g., deodorants–antitranspirants)
Jewelry and ornaments	Food additives (e.g., as colorants or stabilizers)

Total human exposure to aluminum is for the most part the result of uptake from foodstuffs and food additives as well as migration from food contact materials [3].

### Aluminum in foodstuffs

Aluminum occurs naturally in drinking water and in unprocessed foods [12, 15, 17–20]. This natural proportion is also referred to as the “primary content,” and represents the unavoidable aluminum content in foods [16]. Aluminum occurs in foods of both plant and animal origin. The aluminum in plants reflects the content in the soil and in the water resulting from carryover. Certain plants take up more aluminum than others. Examples of plants that preferentially take up aluminum are tea bushes, leafy vegetables, and certain subtropical fruits [14] as well as spice plants [13]. In particular, plants that grow in acidic soil accumulate larger amounts of aluminum. The naturally occurring aluminum concentration in foodstuffs of animal origin is an indication of the aluminum content of the animal feed and the capability of the organs and tissues to accumulate aluminum [14, 21]. Aluminum may find its way into processed foods through food additives as well as through migration from food contact materials that contain aluminum [22].

Table 2 presents typical aluminum concentration levels in foodstuffs of vegetable and animal origin.

**Table 2:** Concentration range of aluminum in foodstuffs. Modified representation of the original from EFSA [21, 27]

Concentration range (aluminum in mg/kg foodstuff)	Foodstuff
≤5.00	Most unprocessed foods
5.00–10.0	Bread, cake, pastries, baking mixes, flour, vegetables: mushrooms, spinach, radishes, chard, lettuce
	Candied fruits
	Animal products: milk products, sausage, offal, seafoods
>10.0	Tea leaves
	Cocoa and cocoa products
	Spices, herbs
	Coffee

It is shown in Table 2 that animal products such as milk products, sausage, or seafood as well as vegetables and baked goods exhibit concentrations of aluminum between 5.00 and 10.00 mg/kg and foodstuffs such as coffee, tea, spices, and herbs are found to have concentrations >10 mg/kg. Concentrations of aluminum in numerous foodstuffs have been determined in the Hessian State Laboratory in previous years and were published in part in the year 2011 [16].

In Table 3 these data are presented, expanded to included results obtained since 2011:

**Table 3:** Aluminum in foodstuffs (mg/kg or mg/L). Modified from Stahl [16]

Product	*n*	Minimum^a^	Maximum^b^	Mean value^c^	Median value^d^
Dates	18	1.23	6.72	3.39	2.57
Pine nuts	9	12.0	38.6	26.1	23.8
Wheat	65	1	19	4	3
Baking mixes	37	1	737	51	6
Bread	107	1	14	3	2
Spelt	28	<BG	3.0	0.63	0.37
Loaf-shaped yeast fruit cakes	60	3	22	10	9
Fine pastries in aluminum trays	38	1	537	19	3
Salt pretzels and similar savory biscuits	185	2	218	13	4
Pasta	24	1	76	10	4
Herbal-teas	12	14	67	40	45
Cocoa powder	37	80	312	165	160
Chocolate	84	6	150	48	39
Confectioneries	115	1	184	17	8
Malt	50	1	12	7	7
Evaporated milk	49	0.08	0.66	0.290	0.205
Soft cheese	13	0.3	5.39	1.68	1.37
Harz cheese	22	0.15	0.78	0.400	0.438
Milk curd	53	0.03	1.73	0.224	0.109
Beer and mixed drinks containing beer, draught beer	237	0.4	4.2	0.5	0.4
Fruit juice and fruit juice drinks	59	0.4	47	3	1
Wine and fruit wine	65	0.4	15	2	1
Mineral water, spring water and table water	171	0.1	0.07	0.01	0.006
Ready-cooked meals in aluminum trays	31	1	13	3	1
Soups	16	1	15	5	3
Pork (canned)	8	0.76	1.35	1.23	1.08
Beef (canned)	6	0.52	1.1	0.634	0.669
Game	149	<BG	1.1	0.110	0.025
Herring (canned)	32	0.16	5.99	1.99	1.60
Crustaceans	45	0.07	40.0	4.47	2.54

*n* number of samples tested

^a^Minimum: lowest values measured in the sample series

^b^Maximum: highest values measured in the sample series

^c^Mean value: arithmetic means of all samples

^d^Median value: median of all samples

As shown in Table 3, the values determined from Table 2 are confirmed by these results. Meat and other animal products such as milk products contain lower concentrations of aluminum than e.g., pine nuts, cocoa powder, chocolate, or bread, including baking mixes.

### Aluminum in or resulting from food additives

Food additives containing aluminum belong to the secondary or anthropogenic sources and may have a decisive influence on the total aluminum concentration in foodstuffs. Various aluminum compounds are approved as additives in the European Union. Elemental aluminum may be used as a colorant. In addition, various additives containing aluminum are allowed for technical use. These include firming agents, releasing agents, and raising agents. The regulation (EC) No. 1333/2008 harmonizes the use of food additives in the European Union. The approved additives and their maximum amounts or maximum concentrations are listed in appendices II and III of the regulation. Considering that aluminum containing food additives may contribute to an increase in the total dietary uptake of aluminum [22], the permissible maximum amounts in these regulations are comparatively high. Table 4 presents a summary of the various forms in which aluminum and aluminum compounds are used as food additives.

**Table 4:** Food additives containing or consisting of aluminum, according to the Austrian Federal Ministry of Health [22]

E-number	Designation	Maximal concentration (mg/L) or (mg/kg)	Limitations	Use
173	Aluminum^a^	*Quantum satis* (The maximum amount is not listed as such. The substances are, however, used according to accepted manufacturing practices, only in amounts that are required to achieve the desired effect and under the condition that the consumer is not misled)	Only external coating of sugar confectionery for the decoration of cakes and pastries	Colorant
520 521 522 523	Aluminum sulfates	200 (expressed as aluminum)	Only candied, crystallized or glacé fruit and vegetables^b^; 520–523 may be added individually or in combination	Firming agent
		30 (expressed as aluminum)	Only egg white^c^	
541	Sodium aluminum phosphate acidic	1000^d^ (expressed as aluminum)	Only scones and sponge wares^d^	Raising agent
554	Sodium aluminum silicate	15,000 mg/kg in the preparation	In fat soluble vitamin preparations	Release agent
		20 mg/kg carry over in cheese	Only for salt intended for surface treatment of ripened cheese, food category 01.7.2^e^	
555	Potassium aluminum silicate	90% relative to the pigment	In E 171 titanium dioxide and E 172 iron oxides and hydroxides	Release agent
556	Calcium aluminum silicate^f^			Release agent
558	Bentonite^g^			Substrate for colorants
1452	Starch aluminum octenyl succinate	35,000 mg/kg in final food	In food supplements as defined in Directive 2002/46/EC due to its use in vitamin preparations for encapsulation purposes only	

Amended according to regulation (EU) No. 380/2012 [39] from 3 May 2012

^a^Category “Other confectionery including breath freshening microsweets” [Category 05.02 of regulation (EC) 1333/2008] [40] period of application: until 31 January 2014

^b^Only candied cherries period of application: from 1 February 2014

^c^Period of application: from 1 February 2014 (E 520 aluminum sulfate) for liquid egg white for egg foams only

^d^Only sponge cakes produced from contrasting colored segments held together by jam or spreading jelly and encased by a flavored sugar paste (the maximum limit applies only to the sponge part of the cake) period of application: from 1 February 2014

^e^Newly certified for this application on 1 February 2014

^f^Authorized until 31 January 2014

^g^Authorized until 31 May 2013

### Aluminum from food contact materials

Aluminum and aluminum alloys are commonly used in the manufacture of utensils that come into contact with food. Along with food additives, these objects represent an additional anthropogenic source of aluminum in food. Before the end of the nineteenth century articles made of aluminum were considered luxury goods; however, with reductions in the cost of production, the scope of application was greatly expanded. Due to the material properties mentioned above, eating and drinking utensils as well as pots and pans have been manufactured out of aluminum since the end of the 1900s. The first widespread application was in the military, for use as eating utensils, pots, and canteens. New procedures for preparation, storage, and packaging of foodstuffs led to increased usage of aluminum in food products. Today, it is impossible to imagine the food sector without aluminum. Table 5 presents a summary of the various uses of aluminum in the field of food contact materials.

**Table 5:** Summary of food contact materials made of aluminum. Source selection from [22]

Food packaging
Aerosol cans (e.g., canned whipped cream)
Aluminum foil
Containers for convenience foods
Lids for yogurt containers
Beverage cans
Coffee capsules
Tubes for mustard, mayonnaise
Packaging for candy
Composite materials for beverage cartons and packaged coffee
Food manufacturing
Aluminum tanks for wine, juices, oil, milk
Baking trays
Meat and sausage hooks
Machine parts
Cooking and kitchen utensils
Baking sheets
Bread boxes
Lids
Coffee pots
Cooking utensils (cooking spoons, ladles)
Pans
Pots
Household equipment
Ice cream makers
Juicers
Moka pots
Coffee percolators

### Specific release limit (SRL)

In September of 2013, the Council of Europe passed a resolution for metals and alloys that come into contact with foodstuffs [23]. In this resolution, specific release limits (SRL) for metals and alloys, including aluminum, were proposed. As a basic principle, the release of metal should remain ‘As Low As Reasonably Achievable (ALARA).’ An SRL specifies, the maximal amount of metal ions (in mg) may be transferred from a defined surface of the contact material to the food (in kg) or food simulant. Known toxicological aspects of the given metals are taken into account. The SRL for the release of aluminum to foodstuffs was specified to be 5.00 mg/kg foodstuff [23]. Data from monitoring programs for foods and food simulants by the food industry as well as from members of the Europe Community show that this value can be adhered to [23]. The SRL is also used by official control authorities. In the studies presented in this paper, consumable liquids were also tested, whereby the SRL for liquids will be presented in the unit mg/L.

### Tolerable weekly intake (TWI)

The Joint FAO/WHO Expert Committee on Food Additives (JECFA) and the Scientific Committee for Food (SCF) published a provisional tolerable weekly intake (PTWI) for aluminum of 7.00 mg/kg body weight (BW) per week. This value was derived from animal experiments studying the toxicology of aluminum in dogs, rats, and mice [24]. According to this, it would be permissible for an adult human weighing 70 kg to take up 490 mg per week and for a child weighing 15 kg an amount of 105 mg per week. More recent animal studies [25] suggest that damage may occur at lower dosages than 7.0 mg/kg per week so that in 2006 the provisional TWI was consequently reduced to 1.0 mg/kg BW. The European Food Safety Authority (EFSA) also specified a TWI for aluminum of 1.00 mg/kg BW per week, based on combined findings of various studies and due to accumulation of aluminum in the body. This represents the present criterion recognized by the European Union [26, 27]. Accordingly, an adult weighing 70 kg can consume 70.0 mg aluminum per week for a lifelong period, 15.0 mg for a child weighing 15 kg.

### Toxicological effects of aluminum

Uptake of aluminum can result in risks to human health. Aluminum has an effect on numerous biological processes in the body. The exact mechanism of aluminum toxicity is, however, not fully understood. It is considered certain that aluminum is potentially cell- and neurotoxic. [22]. Enzyme activity may be disrupted and mitochondrial function may be impaired. Aluminum may also produce oxidative stress [4]. In particular, three organ systems may be negatively affected by aluminum: the hemopoietic system, the nervous system and bones. It has also been suggested that aluminum may play a role in the occurrence of diseases such as breast cancer and Alzheimer’s dementia [22]. The only direct connections that have been shown between aluminum and disease are for hemodialysis encephalopathy, osteomalacia (softening of the bones), anemia, and aluminosis (pathological changes to the lungs) [22, 28]. Of particular importance are aluminum poisoning in the form of encephalopathy and osteomalacia. These effects may occur in patients with chronic kidney disease who are dependent upon dialysis or ingest phosphate binders containing aluminum for extended periods of time [29]. Dialysis encephalopathy^2^ has been brought into connection with increased aluminum concentrations in the brain of dialysis patients. In order to adequately remove phosphate from the blood, the dialysis fluid contains an aluminum compound. The aluminum can then apparently accumulate in the body and in the brain. Patients may suffer from speech difficulties and muscle cramps brought on especially by trivalent aluminum ions, personality changes, accelerated dementia, and depressive moods [30, 31]. The skeletal system has also been suggested as a potential target of aluminum toxicity [28]. Long-term uptake of comparatively large amounts of aluminum, e.g., taking antacids^3^ containing aluminum, may cause an imbalance in the calcium and phosphate household with resultant softening of the bones [32]. Substantial aluminum contamination may result in delayed bone development [31] and development of anemia. Aluminum-induced anemia is typically macrocytic and hypochromatic,^4^ often occurring in dialysis patients. The exact cause of this phenomenon remains unknown [31]. Aluminosis, also referred to as “aluminum lung” is an occupational disease of workers exposed to aluminum and aluminum oxide dust and vapors. Of all aluminum-related health risks, aluminosis has been known the longest. [29]. Aluminum compounds accumulate in the lungs and impair the self-cleaning system of the lungs. In addition, they may elicit inflammatory processes that induce irreversible tissue damage to the respiratory system and the lungs, possibly resulting in fibrosis [33, 34]. Due to inadequate and disputable data, the involvement of aluminum in breast cancer and the development of Alzheimer’s disease as mentioned in the discussion can neither be explicitly substantiated or negated. Indications of a relationship between aluminum and breast cancer result from observations that breast tissue of women with tumors to some extent may exhibit higher aluminum concentrations than tissue from healthy women. It should also be noted that the disproportionate incidence of female breast cancer in the upper outer quadrant rises with year of publication, from 30.9% in 1926 to 43–48% in 1947–1967 and to 60.7% in 1994 [35]. Aside from the improved diagnostic methods for cancer of the breast, the use of antitranspirants or deodorants containing aluminum may also play a role. Since these substances are applied very close to the breast tissue, they could be involved in the development of breast cancer [9]. It must be noted, however, that the data related to this are contradictory and as a consequence it is not possible to draw scientifically tenable conclusions regarding antitranspirants or deodorants containing aluminum and breast cancer. As a reaction to this controversial situation, however, aluminum-free antitranspirants have begun to appear in the market. Alzheimer’s disease is an illness characterized by disorders in cognitive areas such as memory and orientation disturbances [36]. Reports have been made of increased aluminum concentrations in certain areas of the brain [37]. As with the role that aluminum may play in the development of breast cancer, the role of aluminum in Alzheimer’s disease remains very controversial. As possible mechanism, the neurotoxic potential of aluminum and the possibility that aluminum bound to transferrin may enter the brain have been suggested. There is no evidence that aluminum may be a singular causative factor for Alzheimer’s disease, but may act as a co-factor that can abet the development of the disease [22].

### Basis for the study

As a result of the importance of dietary aluminum exposure (see “Aluminum in or resulting from food additives” above) and the common use of aluminum food contact materials (see “Aluminum from food contact materials” above), in particular in private households, the migration of aluminum from contact materials to food was examined. This study was deemed necessary because of the incomplete data on migration of aluminum from household and cooking utensils within the framework of consumer protection and to this extent aluminum drinking bottles and aluminum stove-top moka pots (Part II) as well as aluminum grill pans and camping utensils (Part III) were examined for migration of aluminum to food.

## Methods

To study the migration of aluminum from food contact materials to foodstuffs, various foods and food simulants were examined for their aluminum levels after storage or preparation in food contact materials containing aluminum by means of inductively coupled plasma mass spectrometry (ICP-MS) and inductively coupled plasma optical emission spectroscopy (ICP-OES).

### Samples

A total of 297 samples including food with and without contact to aluminum based cans, bottles, pans, or pots were analyzed. Water for all measurements was tap water from the local supplier (Wasserversorgungsbetriebe Wiesbaden, Germany) according to the guidelines on testing conditions for articles in contact with foodstuffs from the European Commission [38]. The water was stored in polyethylene (PE) canisters at 8 °C in a cold room for 1 week to insure that the same water was used throughout for testing. The naturally occurring background concentration of aluminum in the tap water was 0.7 µg/L, which was negligible compared to the test results (lowest concentration after migration was 10 µg/L), and was subtracted from all measurements of samples prepared with tap water. Powder samples were shaken with water in order to avoid segregation effects. Lumpy food samples were homogenized by means of a Retsch Grindomix GM 200 knife mill (RETSCH GmbH, Haan, Germany). Homogenized samples were solubilized by microwave-based acidic pressure digestion. Two milliliters of deionized water (18.2 MΩ cm), 3 mL of nitric acid (Merck, 65% Suprapur^®^), and 2 mL of hydrogen peroxide (Merck, 30% Suprapur^®^) as oxidizer were added to the samples. A few samples were digested without addition of water. Digestion for two parallel batches each per sample was carried out in an Ultra CLAVE III digestion system (MLS GmbH. Leutkirch, Germany) at 240 °C. The digested samples were diluted to 25 mL with 0.0467 mol/L nitric acid solution.

### Analysis

The aluminum concentration was determined via inductively coupled plasma mass spectrometry (ICP-MS), in accordance with DIN EN ISO 17294-2:2005-02 and inductively coupled plasma optical emission spectroscopy (ICP-OES), in accordance with DIN ISO EN 11885:2009-09. ICP-MS measurement was performed using a NexION 300D (Perkin-Elmer, Waltham, Massachusetts, USA) with baffled Cyclonic Spray Chamber and MEINHARD^®^ Concentric Nebulizers. For the determination of higher aluminum concentrations, the ICP-OES OPTIMA 8300 DV (Perkin-Elmer, Waltham, Massachusetts, USA) with baffled Cyclonic Spray Chamber and Cross-Flow Nebulizer was used. For calibration, commercially obtainable “standard solution for ICP” (multiple elements) by CPAchem (C.P.A. Ltd. Stara Zagora, Bulgaria) was used. Standards and samples were diluted with deionized water (18.2 MΩ cm) and high-purity acids. Rhodium was used as internal standard for ICP-MS measurements. The limit of quantification (LOQ) depended on initial weight as well as on the measurement system (listed in Table 6. Quality assurance was performed by the use of standard reference materials (TM-15.2 water (33.9 ± 4.6 µg/L), rice flour (4.21 ± 0.34 mg/kg), apple leaves (286 ± 9 mg/kg), and blanks, which were also digested and treated the same as the samples. The reference materials were dissolved and analyzed in triplicate, whereby according to our internal specification for quality control, the recovery rate for aluminum had to be between 90 and 110%. In regard to blank values: The chemical blank values (*n* = 3) were found to be 0.005 ± 0.002 mg/kg and are therefore negligible in regard to aluminum concentration in the samples tested.

**Table 6:** Sample materials with initial weight, digestion additives and LOQ

Sample	Initial weight/initial volume	Digestion additive	LOQ
Water	–	–	0.001 mg/L
Oil	0.5 g	HNO_3_, H_2_O_2_, H_2_O	0.1 mg/kg
Citric acid	2.5 mL	HNO_3_, H_2_O_2_, H_2_O	0.02 mg/L
			ICP-MS 3 mg/L ICP-OES
Tea bag and granulated Tea^a^ (instant lemon-tea drink)	5 mL	HNO_3_, H_2_O_2_	0.005 mg/L
Lemon juice	1 mL	HNO_3_, H_2_O_2_, H_2_O	0.025 mg/L
Apple juice with mineral water	5 mL	HNO_3_, H_2_O_2_	0.005 mg/L
Meat filled pasta (ravioli)	1 g	HNO_3_, H_2_O_2_, H_2_O	0.025 mg/kg
Fish	1 g	HNO_3_, H_2_O_2_, H_2_O	0.025 mg/kg
Espresso	5 mL	HNO_3_, H_2_O_2_	0.005 mg/L
Ground coffee	0.5 g	HNO_3_, H_2_O_2_, H_2_O	0.5 mg/kg

*LOQ* limit of quantification

^a^List of ingredients according to the manufacturer: sugar, glucose, acidifier citric acid, cold-water soluble black tea extract 1.28%, vitamin C, lemon extract powder (maltodextrin, lemon juice concentrate), aroma

With regard to the drinking bottles and moka pots please see Part II (Migration of aluminum to food from drinking bottles and moka pots) and for camping articles Part III (Migration of aluminum to food from aluminum bowls and camping utensils).

## Results, discussion, conclusions

All results of this study are presented in Part II (Migration of aluminum to food from drinking bottles and moka pots) and for camping articles Part III (Migration of aluminum to food from camping dishes and utensils made of aluminum).

## Footnotes

1 In 1942, however, it was reported that pteridophytes such as ferns and puzzlegrass need aluminum [6]. Thirteen years later a study was published with evidence that aluminum is necessary for the growth of the tea bush [7].

2 Encephalopathies are functional disorders of the central nervous system [30].

3 Gastric acid binding pharmaceuticals.

4 Microcytic: undersized erythrocytes; hypochrome: erythrocytes are pale (hemoglobin deficiency).
